# Coronaviruses and Central Nervous System Manifestations

**DOI:** 10.3389/fneur.2020.00715

**Published:** 2020-06-23

**Authors:** Mohamed Khateb, Noam Bosak, Maryam Muqary

**Affiliations:** ^1^Department of Neurology, Rambam Health Care Campus, Haifa, Israel; ^2^Department of Psychiatry, Rambam Health Care Campus, Haifa, Israel

**Keywords:** SARS-CoV-2, coronaviral infections, COVID-19, neurological & psychiatric symptoms, brain disorders, neurological infection and inflammation

## Abstract

SARS-CoV-2 is a highly pathogenic coronavirus that has caused an ongoing worldwide pandemic. Emerging in Wuhan, China in December 2019, the virus has spread rapidly around the world. Corona virus disease 2019 (COVID-19), which is caused by SARS-CoV-2, has resulted in significant morbidity and mortality. The most prominent symptoms of SARS-CoV-2 infection are respiratory. However, accumulating evidence highlights involvement of the central nervous system (CNS). This includes headache, anosmia, meningoencephalitis, acute ischemic stroke, and several presumably post/para-infectious syndromes and altered mental status not explained by respiratory etiologies. Interestingly, previous studies in animal models emphasized the neurotropism of coronaviruses; thus, these CNS manifestations of COVID-19 are not surprising. This minireview scans the literature regarding the involvement of the CNS in coronavirus infections in general, and in regard to the recent SARS-CoV-2, specifically.

## Introduction

Coronaviruses constitute a group of single-stranded RNA viruses that cause respiratory, hepatic, enteric, and neurological illness. The severity of symptoms is highly variable, and the range of animal species affected is broad, including humans (1–3). In general, coronaviruses are classified into four major genera: *Alphacoronavirus, Betacoronavirus* (βCoV), *Gammacoronavirus*, and *Deltacoronavirus* (3, 4). Over the last two decades, two novel βCoV strains caused severe human illness (5, 6): the severe acute respiratory syndrome coronavirus (SARS-CoV) and the Middle East respiratory syndrome coronavirus (MERS-CoV). Both strains were associated with high morbidity and mortality rates, and the lack of successful treatments.

In December 2019, the novel severe acute respiratory syndrome coronavirus (SARS-CoV-2) emerged in Wuhan, China. Corona virus disease 2019 (COVID-19) has since become a worldwide pandemic, with high rates of mortality (7–9). The sequence, pathogenesis and cellular entry mechanisms of the SARS-CoV-2 are similar to those of the SARS-CoV (10–12). The major clinical manifestations of SARS-CoV-2 infection are respiratory due to pulmonary complications (13–17). Symptoms can be mild, including fever, headache, cough, dyspnea and myalgia; or severe, such as acute respiratory distress syndrome (ARDS), which sometimes develops about 1 week into the illness and may result in death (7–9, 13–17). Central nervous system (CNS) complications of COVID-19 infection have not been systematically investigated or analyzed.

The neurotropism potential of coronaviruses was demonstrated in previous studies (10, 18). For example, SARS-CoV is believed to enter the brain primarily via the olfactory bulb, resulting in rapid infection with transneuronal spread and minimal cellular infiltration (19). This may cause neural dysfunction especially at the cardiorespiratory centers in the medulla, as was demonstrated in mice transgenic for human ACE2 (20).

Considering the above, we screened the available literature regarding possible neurological manifestations and complications of coronaviruses in general, and of the recent SARS-CoV-2 specifically. Table 1 summarizes human manifestations of SARS-CoV-2 and other coronaviruses in the CNS. Below we describe the evidence of CNS disorders that have been linked with coronaviruses.

**Table 1:** Reports on human manifestations of SARS-CoV-2 and other coronaviruses in the central nervous system.

	**Novel SARS-CoV-2**	**Other coronaviruses [specific strain]**
Meningoencephalitis	Single case report (21)	Several case reports [HCoV-OC43 (22), SARS-CoV (23)]
Acute necrotizing encephalopathy (ANE)	Single case report (24)	
Acute ischemic stroke (AIS)	Several case series (25–28)	
Acute disseminated encephalomyelitis (ADEM)	Single case report (29)	Several case reports [MERS-CoV (30), HCoV-OC43 (31)]
Acute flaccid paralysis (AFP)	Single case report (32)	Single case report [HCoV-229E and OC43 (33)]
Bickerstaff's brainstem encephalitis		Single case report [MERS-CoV (34)]
Anosmia	Several cross sectional studies (35, 36)	

## Subsections According to CNS Disorders

### Encephalomyelitis and Demyelination

Multiple sclerosis (MS) is a chronic inflammatory demyelinating disease of the CNS. The immune reactions involved in MS are attributed to both genetic and environmental factors (37, 38). A possible link between coronaviruses and demyelinating disorders, including MS, has been reported (39–41). Coronavirus strains were suggested to initiate immunopathogeneic events leading to CNS demyelination. Some strains were even proposed as an experimental model for MS to further study the mechanisms of virus-induced demyelination (42–44). In animals, the murine coronavirus JHM was demonstrated to cause demyelination (45). The ability of coronaviruses to produce demyelination in experimentally infected mice has led to studies that investigate the possibility of human coronaviruses in MS brain tissues. Interestingly, nucleic acids of the human coronavirus 229E strain were detected in the CNS tissue of four of eleven MS patients, indicating neurotropism of the 229E strain (46). Moreover, coronavirus-like particles under electron microscopy were found in autopsies from two MS patients (47). Murray et al. (48) demonstrated that direct intracerebral administration of coronaviruses might cause demyelinating disease in primates. Using *in situ* hybridization, they detected coronavirus RNA sequences in the brain and in demyelinating plaques in 12 of 22 MS patients (49). In contrast, other studies that used polymerase-chain-reaction (PCR) with specific primers for the two human coronaviruses 229E and OC43 did not show any difference between the MS and the control groups in coronaviral RNA detection in brain tissues (41, 50). Moreover, antigenic assessment by comparing levels of coronavirus antibodies failed to support the claim of coronaviruses as an etiology for MS. This is because no significant differences were found between samples of MS patients and control subjects (51).

Multiple mechanisms were proposed to explain demyelination by coronaviruses in animal models. JHM virus-induced demyelination was largely correlated with cytopathogenic properties of the virus for oligodendrocytes (52). The molecular basis of this process was linked to the E2 sub-region (45). Additional mechanisms subsequently emerged. One of them involves molecular mimicry between coronaviruses and myelin as a basis for the autoimmune reaction leading to cross-reactivity of T-cells (53, 54). Furthermore, RNA recombination demonstrated that the S gene of the coronavirus mouse hepatitis virus (MHV) is related to certain molecular aspects of demyelination. This indicates the potential role of viral envelope S glycoproteins in autoimmune-induced demyelination (43). Mutations in the spike glycoprotein of human coronavirus OC43 (HCoV-OC43) modulated the disease from chronic encephalitis to flaccid paralysis and demyelination (55). Other studies emphasized the importance of T cells, especially CD8, in the process of demyelination in mice infected with coronaviruses (56, 57). Accordingly, gamma-interferon was shown to lead to demyelination mediated by CD8 cells. Nevertheless, Kim et al., showed that the specific antibodies against the coronavirus JHM in animal models were sufficient to induce demyelination in the absence of T cells. They also showed that the destruction of myelin in these cases resulted from the complement system, as well as from fc receptor-dependent mechanisms (58, 59). Additional mechanisms related to chemokines like CXCL10 and chemokine receptor CCR5 were also described (60, 61).

Coronaviruses have been associated with other demyelinating pathologies like acute or subacute disseminated encephalomyelitis (ADEM) in humans (31, 62). ADEM is a rare acute inflammatory demyelinating disease that may follow viral infections. Intracerebral inoculation of the human coronavirus HCoV-OC43 into BALB/c mice caused acute encephalitis with cellular death by necrosis and apoptosis (63). Several months of follow-up of viral RNA led to the conclusion that viral persistence could be associated with increased neuronal degeneration, resulting in neuropathology and motor deficits. In line with these experimental findings, in a child who presented with ADEM, cerebrospinal fluid (CSF) tested positive for HCoV-OC43, using PCR (31). Several patients infected with MERS-CoV were reported to demonstrate a severe neurological syndrome; one of them met the criteria for ADEM (30). Another report described a patient who was diagnosed with the presumably post/para-infectious syndrome of Bickerstaff's brainstem encephalitis (34). Recently, a link between SARS-CoV-2 infection and ADEM was described in one woman (29).

### Optic Neuritis

Acute monosymptomatic optic neuritis (AMON) is sometimes an initial symptom of MS; the implication of coronaviruses in the etiology of MS is described above. However, a study that compared the presence of coronavirus RNA in the CSF of individuals with AMON and controls revealed no significant differences (64).

### Viral Meningoencephalitis

Encephalitis is a serious and potentially fatal neurologic illness characterized by CNS inflammation. The etiologies of the majority of those affected are viral, yet identifying the specific causal organism can be difficult. Morfopolu et al., described an 11-month-old boy with severe combined immunodeficiency who had symptoms of acute encephalitis. The conventional diagnostic PCR assay was negative. Eventually, using three independent methods, the HCoV-OC43 in the child's brain tissue was identified (22). Clinical presentation suggestive of encephalitis was also reported in a 59-year-old woman infected with SARS-CoV (23). The patient developed status epilepticus, which followed a few hours of disorientation during hospitalization in the intensive care unit (ICU) due to respiratory symptoms. CT scan of the brain did not detect any abnormality. CSF did not contain any cells but was positive for SARS-CoV using real-time PCR analysis.

One of the early symptoms of COVID-19 infection is headache. The first documented patient with meningoencephalitis as a result of SARS-CoV-2 infection had neck stiffness and transient generalized seizures (21). SARS-CoV-2 RNA was not detected in the nasopharyngeal swab but was detected in his CSF.

### Parkinson's Disease

Parkinson's disease is a common neurodegenerative disease that primarily affects the basal ganglia system. Disruption of motor functions is one of its main clinical manifestations. CSF seropositivity for several strains of coronaviruses has been reported in Parkinson's disease (65). However, this correlation was not confirmed or replicated over nearly three decades.

### Acute Flaccid Paralysis

Acute flaccid paralysis (AFP) is a life-threatening syndrome characterized by dramatic weakness in muscles, often respiratory and bulbar muscles, as a result of damage to lower motor neurons. When secondary to peripheral nerve involvement, the syndrome is usually a manifestation of Gullian–Barre syndrome. The latter was reported to be related to MERS-CoV (34), and also to SARS-CoV-2 (66), but is out of the scope of the current review of CNS involvements. In other cases of AFP, the paralysis is due to damage at the level of the anterior horn of the spinal cord, and termed acute flaccid myelitis. In such cases, other etiologies, especially various infections, were described (67). A similar clinical picture can be secondary to acute myelopathy, most commonly the result of inflammatory diseases of the spinal cord, either as part of ADEM presentation or isolated acute transverse myelitis.

Turgay et al., described a 3-year-old girl who was admitted with lower respiratory tract infection and AFP, in the presence of normal nerve conduction studies and electroneuromyography (NCS-EMG) and CSF content. Magnetic resonance imaging (MRI) was unavailable in the managing center. Co-infection with human coronaviruses 229E and OC43 was detected by real-time PCR analysis of nasal swab samples (33). Interestingly, the girl showed clinical improvement after treatment with IVIG. Similarly, a 66-year-old man who presented with AFP and urinary and bowel incontinence, which developed about 1 week after fever and subsequent respiratory symptoms, was eventually diagnosed with COVID-19 (32). No CSF, NCS-EMG, or spinal MRI were obtained due to pandemic-related reasons, but the authors emphasized that the sensory clinical features implied spinal involvement. Following treatment that combined antibiotic agents, anti-viral drugs, dexamethasone and IVIG, the patient exhibited clinical improvement within a rather short follow-up period.

### Anosmia

Loss of appetite has been reported in many patients with COVID-19; and anosmia (loss of the sense of smell) is recognized as a common and early symptom (10, 18, 35). As previously mentioned, SARS-CoV-2 has neuro-invasive propensity, which has been demonstrated as a common feature of coronaviruses. This supports the possibility that the virus infects the olfactory bulb in the early stages, and progresses from there to various brain regions in later stages.

### Disruption of Central Respiratory Control

The exact route by which SARS-CoV-2 enters the CNS is still poorly understood. Coronaviruses have been reported to first invade peripheral nerve terminals, eventually reaching the CNS via a trans-synaptic transfer (18, 68). Viral antigens have been detected in brainstem nuclei such as the nucleus of the solitary tract and nucleus ambiguus (8, 10, 69). This indicates possible dysfunction of the cardiorespiratory center in the brainstem as a complication of coronaviruses infection. Disruption of the function of CNS respiratory centers in the brainstem is believed to exacerbate respiratory failure and impair the state of consciousness of individuals infected by coronaviruses.

### Acute Necrotizing Encephalopathy (ANE)

ANE is a rare complication of viral infections like influenza, which may be caused by intracranial cytokine storms that break down the blood-brain-barrier (70). Evidence from the COVID-19 pandemic suggests that for a subgroup of patients with severe presentation, this condition might be complicated with cytokine storm syndrome (71). Radiological diagnosis of ANE was reported in a woman with COVID-19 who presented with altered mental status (24).

### Acute Ischemic Stroke (AIS)

Accumulating evidence implies a possible link between infection with the novel SARS-CoV-2 and acute ischemic stroke (AIS). In general, infectious diseases of the respiratory system are a possible and independent risk factor for plaque rupture that results in AIS (72, 73). The cytokine storm caused by coronavirus infection increases the risk of thrombosis in general, and of AIS specifically (9, 71, 74). Moreover, changes in platelet count and D-dimer were reported in CoV-SARS2 infection (9). An Italian study showed an increase in the ratio of arterial as well as venous thromboembolic events in patients with COVID-19 (75). Of 388 patients in that study, AIS presented in nine (2.5%). Interestingly, the reason for hospitalization in six of the nine patients was the neurological presentation of AIS. An observational study of 184 patients hospitalized in the ICU with COVID-19 demonstrated that five of the surviving 78 had AIS (25). Similarly, a case series was published of five SARS-CoV-2 patients presenting with acute AIS secondary to large vessel occlusion (LVO) (26). All five patients were younger than 50 years old, two of them were without risk factors for stroke. In another case series of COVID-19 patients with AIS during their hospitalization (27), all six had risk factors for stroke, and were detected with LVO. Interestingly, three of the six patients demonstrated cerebral ischemia in vascular multiple territories. Notably, five of these patients had been positive to lupus anti-coagulant. This supports previous studies that suggested that infection with SARS-CoV-2 triggered production of antiphospholipid antibodies (76). In a case series of four patients with COVID-19 who presented with AIS, two of them had LVO of the middle cerebral artery (28). Taken together, the evidence suggests that infection with coronaviruses might increase the risk of AIS.

### Other Neurological Manifestations

Additional neurological signs, reported in patients with severe SARS-CoV-2 with ARDS include myalgia (29, 77), diffuse corticospinal tract signs with enhanced tendon reflexes and ankle clonus, and dysexecutive syndrome (78). Interestingly, in the study that described the latter manifestations, imaging findings included perfusion abnormalities in all the patients, leptomeningial enhancement in most of them, and cerebral ischemia in about 23% (78). However, these results should be interpreted cautiously, as they may represent, at least in part, different effects of the critical illness state, regardless of the SARS-CoV-2 etiology.

### Psychiatric Sequels

In general, the evidence available on the direct relations between respiratory neurotropic viruses and psychiatric disorders is relatively sparse. Specifically, a positive association was described between infection with coronaviruses (NL63 strain), as reflected by seropositivity, and mood disorders (79). An additional serological study demonstrated a rise in the level of antibodies against the same strain of coronaviruses in individuals with recent onset of psychosis (80).

Early reports from the ongoing COVID-19 pandemic suggest psychiatric morbidities that have some resemblance to those reported in the early phase of the SARS outbreak. Patients are reported to experience fear, loneliness, anger and general distress, which have been primarily attributed to the need for quarantine; and infection symptoms such as fever and cough (81). Moreover, adverse effects of treatments such as insomnia from corticosteroids, and psychotic disorder from chloroquine, might exacerbate patients' mental status (82, 83). Additional reasons for psychiatric morbidities include experiencing adverse effects of treatment during hospitalization, uncertainty regarding prognosis and undergoing ICU care. Therefore, infection with of SARS-CoV-2 is considered a traumatic experience; this enhances anxiety and substantial mental distress.

In the immediate aftermath of the SARS epidemic, various psychiatric morbidities were reported. These included depression, adjustment reactions, anxiety, agitation, psychotic symptoms, delirium, and even higher suicidality (82, 84, 85). In the early recovery phase, up to 35% of SARS survivors showed signs of anxiety, depression or both (84, 86). Importantly, post-traumatic stress reactions were identified in SARS survivors in the early, 2–4 week period, following discharge. Furthermore, about 45% of the respondents in that outbreak were diagnosed with at least one psychiatric disorder during the study period. Psychiatric disorders such as major depression, post-traumatic stress disorder (PTSD) and adjustment disorder were described during the 6 months after patients' discharge (84, 85). Cumulative incidence of any psychiatric disorder during the 3 years following the SARS outbreak was reported to reach 59% in SARS survivors (86). For example, the cumulative incidence rates for PTSD and depressive disorders were about 48 and 44%, respectively (86). Similarly, the prevalence of psychiatric morbidity at 30 months after SARS infection was reported to reach up to one-third of the total survivors who presented with signs of various psychiatric diagnoses. At that timepoint, about 26 and 16% had PTSD and depressive disorders, respectively (86).

The above highlights the urgency of addressing mental health care needs related to the current pandemic of COVID-19, particularly for those with comorbid mental disorders. Lessons learned during the SARS outbreak should be applied to designing strategies in the novel COVID-19 outbreak (87).

## Discussion

Accumulating evidence from the current SARS-CoV-2 pandemic, together with literature on other coronaviruses, suggest that infection with coronaviruses may be related to CNS manifestations or complications, including anosmia, acute ischemic strokes, viral meningoenchephalitis, acute necrotizing encephalopathy, acute flaccid paralysis, and other presumably post/para-infectious syndromes. In addition, patients with COVID-19 may be particularly prone to mental disorders including PTSD, major depression and adjustment disorder. Therefore, we believe these patients should be carefully monitored for clinical signs suspecting CNS involvement such as headache, seizures, sensory or motor deficits, and altered mental state. Early diagnosis of CNS manifestations in individuals with COVID-19 may be crucial for their overall prognosis. A multidisciplinary approach should be emphasized by health authorities, which would provide urgent mental health support for the affected, and also for health care workers.

## Author Contributions

MK initiated the idea, screened the available literature about neurological manifestations in coronavirus infections, created and designed the manuscript. MM created the section on psychiatric sequels of coronavirus infections and contributed to the final revisions. NB contributed to the overall manuscript design. All authors contributed to the article and approved the submitted version.

## Conflict of Interest

The authors declare that the research was conducted in the absence of any commercial or financial relationships that could be construed as a potential conflict of interest.
